# Carcinoma in Women

**Published:** 1902-09

**Authors:** 


### Carcinoma in Women.

PROFESSOR WILLIAM JAPP SINCLAIR, of Manchester, chairman of the section on obstetrics and gynecology, at the recent meeting of the British Medical Association, chose for the subject of his address "Carcinoma in Women, chiefly in its clinical aspects." This important subject, as was to be expected, was most ably dealt with, and the large audience assembled to hear the distinguished speaker testified again and again its approval when he made telling points, as frequently happened.

In regard to the pathology of cancer Prof. Sinclair thought little or nothing had been accomplished in solving the question of cause—and the cause of the disease is, after all, the most important problem, next to prevention, that remains to be solved; and, indeed, both go hand in hand, for the cause once discovered, prevention will be simplified. If pathology has been without result, not so with surgery in the opinion of the speaker. Although he quoted some very gloomy views expressed at recent gynecological meetings in this country and Germany, and he very aptly said that a wave of mental depression had been passing over the gynecological world. For himself, however, he did not share their pessimistic views, and proceeded to point out that the results of the best known operators continue to improve, thus offering great encouragement for persistent future efforts. They were obtaining, he said, better immediate results, and the percentage of immunity for five years after operation—the accepted test—was growing.

Not only this, but the percentage of operable cases when first seen had also increased, since patients came earlier under observation, while improvements in technic had extended the indications for treatment. Statistics of carefully reported work done under circumstances in which sources of error were reduced to the minimum were pointed to in proof of these assertions.

Professor Sinclair said that one of the evil results of the depression arising from the unfavorable consequences of vaginal hysterectomy had been to resort, in many instances, to the extreme of dissecting out the whole of the lymphatics and cellular tissue of the pelvis along with the internal sexual organs, the impression being that the chances for better remote results thereby would be increased. He characterised this radical method of operating as little better than homicidal vivisection, which to his mind were unjustifiable. He did not believe that cancer of the uterus was on the increase, but, on the other hand, the disease in realty was diminishing. He counseled no relaxation in the efforts to discover the cause of cancer or to improve the technic of its surgical management.

Dr. Sinclair asserted that cancer was infectious to the individual already suffering from the disease but not to others; hence the remedy lay in early surgical intervention. In this address it had been his endeavor to direct attention to the importance of exact scientific clinical methods of observation. In pursuance of this object he deemed it necessary to direct specific attention to the futility of much that has been done in the past to establish the etiology and true pathology of cancer. He welcomed all honest enlightened effort and deprecated none. It was his hope that in this research every general practitioner of medicine would coöperate with those engaged in special lines of work. The labors of both sets of workers should be complementary. The family physician generally knew most about the history and environment of the patient and could obtain relative imformation that often was denied to the specialist.

Concluding, he said it was his earnest hope and desire that some of the younger men with more energy, better opportunities, starting on a higher level of knowledge, would professedly take up this great enterprise and see whether by calm, industrious and enlightened clinical observation, alone or collectively, they could not discover the cause and the remedy for this mysterious and dreadful malady.

Running all through this notable address is that dignity of expression and earnestness of purpose so characteristic of the English nature, and it needs must be read to be appreciated. We have made this somewhat extended résumé of the paper, because the subject it discusses is of transcendent importance, and because further, of the strong, though somewhat unique, fashion the distinguished chairman employs in presenting it; and, because, still further, it so nearly expresses views that we have been approaching for some time past.

